# Nickel nanoparticle-activated MoS_2_ for efficient visible light photocatalytic hydrogen evolution†

**DOI:** 10.1039/d2nr01489k

**Published:** 2022-04-28

**Authors:** Xinying Shi, Meng Zhang, Xiao Wang, Andrey A. Kistanov, Taohai Li, Wei Cao, Marko Huttula

**Affiliations:** Nano and Molecular Systems Research Unit, University of Oulu P.O. Box 3000 FI-90014 Oulu Finland andrey.kistanov@oulu.fi; School of Physics and Electronic Engineering, Jiangsu Normal University Xuzhou 221116 China; Department of Physics, East China University of Science and Technology Shanghai 200237 China mzhang@ecust.edu.cn; College of Chemistry, Key Lab of Environment Friendly Chemistry and Application in Ministry of Education, Xiangtan University Xiangtan 411105 China

## Abstract

Direct sunlight-induced water splitting for photocatalytic hydrogen evolution is the dream for an ultimate clean energy source. So far, typical photocatalysts require complicated synthetic processes and barely work without additives or electrolytes. Here, we report the realization of a hydrogen evolution strategy with a novel Ni–Ag–MoS_2_ ternary nanocatalyst under visible/sun light. Synthesized through an ultrasound-assisted wet method, the composite exhibits stable catalytic activity for long-term hydrogen production from both pure and natural water. A high efficiency of 73 μmol g^−1^ W^−1^ h^−1^ is achieved with only a visible light source and the (MoS_2_)_84_Ag_10_Ni_6_ catalyst, matching the values of present additive-enriched photocatalysts. Verified by experimental characterizations and first-principles calculations, the enhanced photocatalytic ability is attributed to effective charge migration through the dangling bonds at the Ni–Ag–MoS_2_ alloy interface and the activation of the MoS_2_ basal planes.

## Abstract 2

Direct sunlight-induced water splitting for photocatalytic hydrogen evolution is the dream for an ultimate clean energy source.

## Graphical abstract

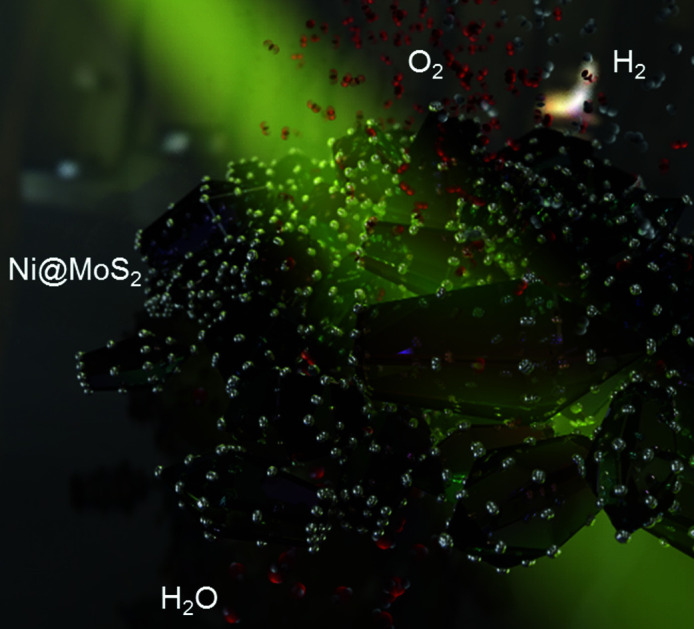

## Introduction

Since the inception of water photolysis,^1^ the dream has been to convert solar energy into hydrogen using only sunlight, water, and cheap catalytic media, serving as an ultimate solution for energy and environmental sustainability.^2,3^ This still remains unachieved due to limited H_2_ production efficiency,^4–6^ high reagent costs, and complicated material synthesis.^6^ Although capable of water splitting, present semiconductor photocatalysts are only active with additives or high energy photons for photochemical hydrogen evolution.^7–11^ The sunlight hydrogen evolution reaction (HER) requires a suitable bandgap from the semiconducting catalyst to facilitate the absorption of visible light and provide proper activation energy for water oxidization. Undesirable electron–hole recombination should be delayed or suppressed so that the photogenerated electron–hole pairs can actively participate in the redox reaction of water.^12^ These stringent conditions may be simultaneously satisfied on a heterojunction composite where a semiconductor matrix and an electron reservoir are interconnected through a tunnel for irreversible charge transfer.

Among the various host candidates, layered MoS_2_ is endowed with unique optical and electronic properties, moderate bandgaps as well as immense possibilities for designing structures and functionalities.^13,14^ The edge sites of monolayer MoS_2_ have been found to be active for HER and can be engineered by various routes to increase the catalytic efficiency.^15–18^ Enhancements of the overall HER performance have been reported on defective monolayers with sulfur vacancies or grain boundaries on the basal planes.^19–21^ In practice, experimental^22,23^ and theoretical^24–26^ efforts have been dedicated to activating the inert basal planes of monolayer MoS_2_ by introducing S vacancies. However, mass production of these catalysts is limited by the required lab conditions and prerequisite preparation of the monolayers. In fact, multilayer MoS_2_ is also capable of HER after appropriate treatments.^27^ As a cheap naturally occurring mineral, molybdenite exhibits competitive electron mobility with its monolayer counterpart.^28^ The electronic and chemical properties of MoS_2_ flakes can be tuned *via* metal nanoparticle doping,^29^ for example, along with possibilities to host rather large-sized dopants *via* edge contact.^30,31^ Effectively joined multilayer MoS_2_ and nickel nanoparticles (NiNPs) exhibit promoted electrical and photocatalytic performance.^31^ The relatively large size of the metal nanoparticles is also beneficial to further applications, *e.g.*, constructing transistors by depositing conductive lines.

Here, we report an efficient H_2_ production strategy under visible light with the help of metal nanoparticle-decorated MoS_2_ multilayers. Nickel nanoparticles are attached by the Ag nanobuffer to the semiconducting MoS_2_, serving as reservoirs for photoinduced electrons and reduction sites for protons. With a sustainable H_2_ production rate of 73 μmol g^−1^ W^−1^ h^−1^ with the reported mass-producible photocatalysts, the present work provides an eco-friendly and industrially scalable path for future clean and sustainable fuel production.

## Results and discussion

### Morphology

Despite the unique hosting ability of multilayer MoS_2_, the design and selection of the joining counterparts and the synthetic route require arduous efforts. Here, we connect Ni nanoparticles to MoS_2_*via* the Ag nanobuffer. Compared to that of MoS_2_, the lower work functions of Ni and Ag reduce the energy barrier at the metal–semiconductor (M/S) interface. Nickel and its oxide/hydroxide are widely used for H_2_ storage and water splitting due to their intrinsic affinity to protons and switchable oxidization states.^32^ This may help the overall HER efficiency. The synthesis was carried out through a one-step wet chemical method. This facile route employed ultrasound to disperse and mix reagent powders and supply reaction energy through local cavity formation.^33^ The reaction was performed in a AgNO_3_ aqueous solution where Ag^+^ could react as a common reagent with both Ni and MoS_2_.

**Fig. 1:**
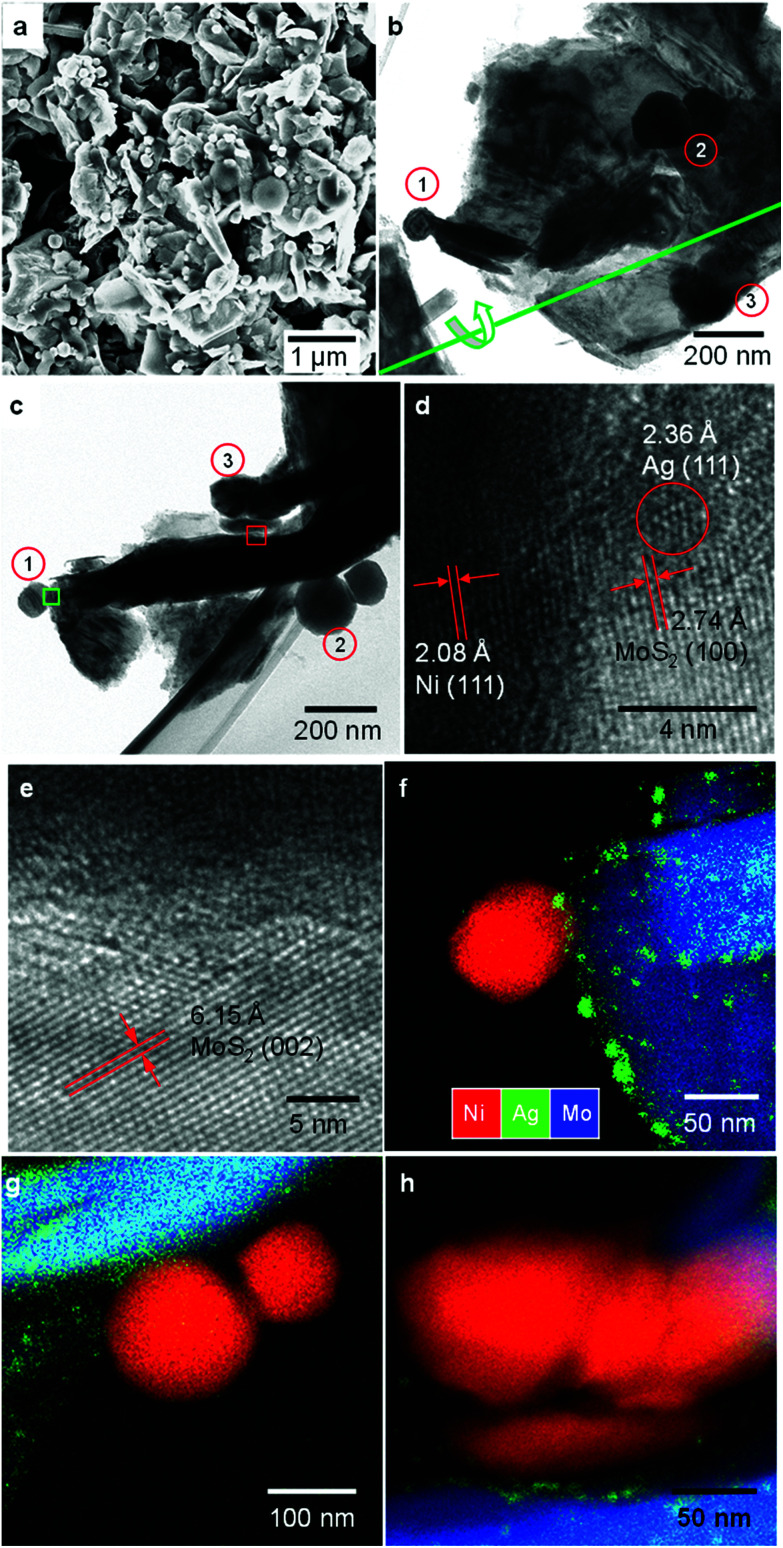
Morphological and elemental characterizations of the prepared composites. (a) SEM image of the catalyst. (b) TEM image presenting the size and distribution of Ag and Ni on MoS_2_ matrices. Three NiNP regions are assigned with numbers. The green line denotes the axis of the TEM specimen holder. (c) The same region as panel (b) with a tilt angle of 62°. (d–e) HRTEM images of interfacial regions marked with green and red squares, respectively, in (c). (f–h) TEM-EDS mappings of regions 1, 2, and 3, respectively.

The typical morphology of the Ni–Ag–MoS_2_ composite is presented in Fig. 1. The general survey in Fig. 1a shows that a large amount of NiNPs are firmly attached to the MoS_2_ substrate. The elemental abundance of the as-prepared sample agrees well with the stoichiometric ratio given by energy-dispersive X-ray spectroscopy (EDS), shown in Fig. S1.† In addition, Ni and MoS_2_ do not tend to approach each other without silver's participation (Fig. S2†). The detailed morphological arrangement within the Ni–Ag–MoS_2_ composite was investigated *via* transmission electron microscopy (TEM) and depicted in Fig. 1b–h. Several NiNPs can be anchored to one MoS_2_ flake, as illustrated in the front view (Fig. 1b) and side view after tilting the sample holder by 62° (Fig. 1c). The flake affords two possible contact modes to the NiNPs: edge contact at region 1 and basal connections at regions 2 and 3. High-resolution TEM (HRTEM) reveals the lattice orientations at the Ni and MoS_2_ interface. The lattices shown in Fig. 1d are indexed to the matrix MoS_2_ (100), buffer Ag (111), and Ni (111) planes. Non-crystallized features are observed in the figure along with the lattice fringes. Thus, the foreign Ni nanoparticles are connected to the layered host through edge contact *via* either non-crystallized content or crystalized Ag (111) nanoparticles. As shown in Fig. 1e, the outermost site of a NiNP is attached to the MoS_2_ (002) basal plane by the Ag buffer. This demonstrates successful metal decoration onto the inert basal surface. The EDS mappings in Fig. 1f–h clarify the Ag distributions on both the edge sites and basal planes of MoS_2_. The mixtures of Ni, Ag and Mo in Fig. 1f demonstrate the formation of a ternary alloy composed of Ni, Ag and MoS_2_, which was previously denoted as amorphous content. The Ag buffers are intercalated between the Ni and MoS_2_ planes, as shown in Fig. 1g and h. Unlike the nanocrystallized form at the matrix edges, rather thin Ag buffers reside on the plane and seem to form discontinuous grains, as seen in Fig. 1h. No element mixture is visible at the Ni–MoS_2_ interface. Thus, Ag behaves as an intercalated layer between the semiconductive and metallic partners.

The general microstructure and porosity of the semiconductor and the metal particles are retained after synthesis, as proved by their X-ray diffraction patterns (Fig. S3†) and the Brunauer–Emmett–Teller (BET) test (Fig. S4†). The specific surface area of the synthesized composite is around 13.62 m^2^ g^−1^, agreeing with the value from multilayer MoS_2_ samples.^34^

### Electrical performance

**Fig. 2:**
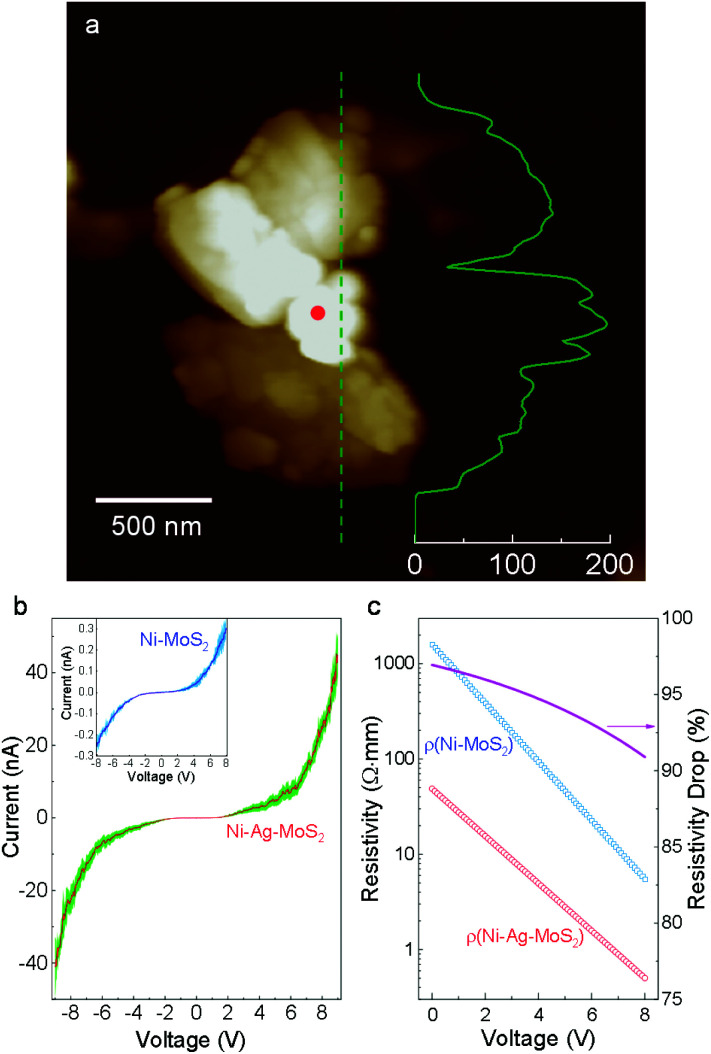
Electronic properties. (a) AFM profile of a typical Ni–Ag–MoS_2_ sample. The inset curve shows the height profile along the denoted line (scale bar in nm). (b) *I*–*V* curves obtained from Ni–Ag–MoS_2_ and Ni–MoS_2_ samples. The shadings represent standard deviations. (c) Electrical resistivity calculated from the *I*–*V* curves.

Charge migration in semiconductors is critical for photocatalytic performance. However, the heterojunction normally suffers from large electrical resistivity at the interface, lowering the water splitting efficiency.^12^ Here, we evaluate the contact mode and junction resistivity by performing current–voltage (*I*–*V*) measurements on the nano-scale Ni–Ag–MoS_2_ composite. Morphological and elemental information is collected and employed as a reference for precise control of the top positions in conductive atomic force microscopy (AFM). As demonstrated in Fig. S5,† a closed circuit is formed among the AFM tip, Ni–Ag–MoS_2_ composite, the gold-plated substrate, and the AFM machine. The *I*–*V* measurements are carried out on an isolated sample, as shown in Fig. 2a, whose elemental distribution and quantification were pre-examined using SEM and EDS (Fig. S6†). The height profile curve demonstrates that the Ni particle is not connected to the metallic substrates.

Fig. 2b plots the *I*–*V* curves obtained from this sample and from a direct Ni–MoS_2_ contact without any noble metal buffer.^31^ Under the same voltage, the current is much larger through Ni–Ag–MoS_2_ compared to its physically contacted counterpart. The calculated resistivity shown in Fig. 2c demonstrates a drastic reduction of the electric resistivity in the low-voltage region. In the high-voltage region, the resistivity of Ni–Ag–MoS_2_ declines slower than that of the sample without the Ag buffer. This is expected since metallic contact between a M/S interface leads to more stable resistivity than a loosely conductive M/S Schottky barrier under higher voltage.^35^ The Ni–Ag–MoS_2_ composite is electrically conductive with easier charge flows.

### Photocatalytic water splitting

**Fig. 3:**
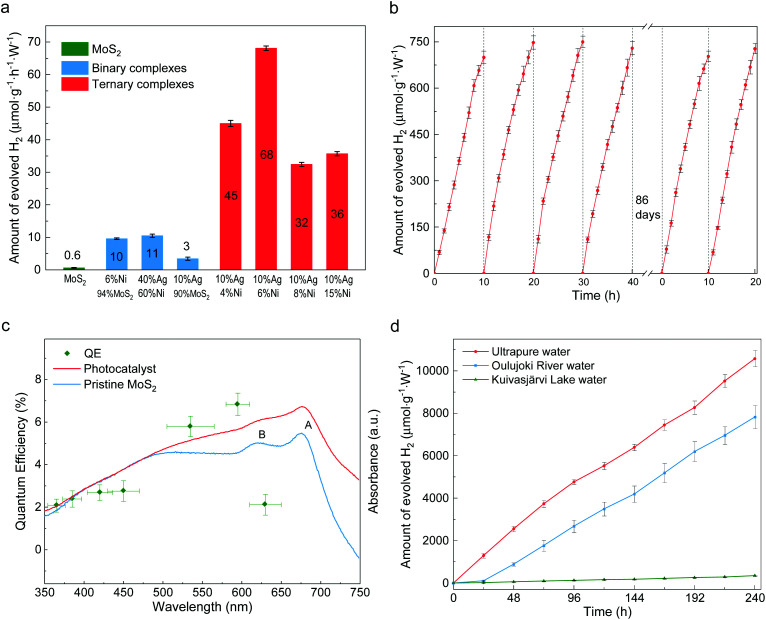
Photocatalytic water splitting ability of the catalysts. (a) Water splitting ability of photocatalysts with different compositions. The ternary composites shown in red are comprised of 10% silver, 4–15% nickel and the remaining amount of MoS_2_. (b) Time course of hydrogen evolution under visible light irradiation (*λ* > 400 nm). (c) Wavelength dependent absorbance and QE of the synthesized photocatalyst and pristine MoS_2_. Horizontal error bars represent the full width at half maximum (FWHM) of the LED light spectra and those in vertical directions come from discrepancies among repeated experiments. (d) Long-term continuous hydrogen production from pure water and natural water. Error bars in panels (a) and (b) were obtained from repeating measurements three times, those in panel (d) from two repeats, and in (c) from the error propagation of the QE formula.

The photocatalytic activity for hydrogen evolution was evaluated under visible light irradiation without any additive. The incident visible light irradiation (*λ* > 420 nm, Fig. S7†) was emitted from LED lamps of 0.495 W. First, the composition impacts on HER rates were studied. As shown in Fig. 3a, different ternary compounds were employed as photocatalyst along with control groups of pure MoS_2_ layers and binary complexes of Ag-decorated Ni and Ag-decorated MoS_2_. The pristine multilayer MoS_2_ can barely split water,^36^ while slightly enhanced HER abilities are found in the cases of Ag_40_Ni_60_ and (MoS_2_)_90_Ag_10_ as a result of plasmon-resonance absorption.^37–39^ Unlike the inert pristine MoS_2_ and binary compounds, all the ternary composites possess good photocatalytic abilities. A maximum value of production efficiency is reached with the sample loaded with 10 wt% Ag and 6 wt% Ni. A time course of hydrogen evolution with the optimal catalyst of (MoS_2_)_84_Ag_10_Ni_6_ is shown in Fig. 3b. It delivered an average HER rate of 73 μmol g^−1^ W^−1^ h^−1^ during the first 6 reaction cycles, with each cycle experiencing 10 hours of visible light irradiation. After 86 days of storage in water in ambient conditions, the photocatalyst preserved its activity without functionality decay, indicating that the catalyst is stable and resistant to photocorrosion. The present ternary composites also have better HER efficiency than their auric peer MoS_2_–Au–Ni, in which the joint interfaces are established through the edge contact mode,^31^ as shown in Fig. S8.† The enhanced efficiency is mostly attributed to the activation of the MoS_2_ basal plane.^22^

We also investigated the quantum efficiency (QE) of HER for the MoS_2_–Ag–NiNPs systems. The UV–visible absorbance spectra are studied and plotted in Fig. 3c for the (MoS_2_)_84_Ag_10_Ni_6_ photocatalyst. Inherited from the multilayer MoS_2_, the synthesized Ni–Ag–MoS_2_ photocatalyst keeps the semiconducting band feature (Fig. S9†). Compared to the host, the catalyst shows quenched excitonic peaks of A and B due to the introduction of transition metals.^40,41^ However, the absorbance over 475 nm is significantly enhanced, indicating a stronger photoexcitation of electrons from the lower energy bands to the lower sites of the conduction bands. The QE is evaluated in a uniformly dispersed aqueous suspension under magnetic stirring (Fig. S10†). In general, the QE follows the absorbance trend of the synthesized photocatalyst. It increases from 350 nm, reaches 7% at 595 nm and then drops. The photocatalyst stays reactive in nearly the whole visible wavelength range and shows potential to overcome the drawback of single photocatalysts (*e.g.*, g-C_3_N_4 _^42–44^) in comparison with their Z-scheme counterparts.^45^

The photocatalysis is durable in time and versatile in ambient conditions. A 10-day continuous water splitting experiment resulted in a hydrogen production rate of 44 μmol g^−1^ W^−1^ h^−1^ from pure water (red line in Fig. 3d). It reaches as much as 63% of the short-term performance shown in Fig. 3b and the decrease of reactivity is attributed to the accumulated H· radicals in the liquid, which hinder the reduction of H^+^. The catalyst can also split natural water, *e.g.*, river water (Oulujoki River, Oulu, Finland) and lake water (Kuivasjärvi Lake, Oulu, Finland). It thus provides a promising prospect for direct sunlight HER in natural water bodies. There is an obvious decrease in natural water splitting compared to that of ultrapure water, *e.g.*, a 26% decrease for river water. Such a decrease is ascribed to the colored organic contents. These organic dyes could be simultaneously decolorized during water splitting, suggesting possible water purification capability. The total organic carbon (TOC) measurement of lake water shows that the TOC level drops from 14.1 ppm to 9.65 ppm and the original color was fully removed (Fig. S11†). Although the degradation process usually consumes photoexcited holes, reactions in natural water may be complicated due to the existence of multiple unknown organic species. This may lead to multi-step redox reactions, in which both electrons and holes may be consumed, making the degradation a competing process with water splitting.^46,47^ Besides, it can be seen from Fig. S12† that the densely colored water absorbs more photons, such that less of them can be utilized by the photocatalysts. It can be also seen that much less hydrogen is produced from river and lake water during the first day than on average, caused by the competing decoloration process. In addition to lab tests, solar water splitting was also achieved under sunlight irradiation. As shown in Fig. S13,† the HER can be triggered by indoor sunlight and H_2_ was continuously produced for months.

We also studied the chemical stability of the catalysts. First, elemental quantification was carried out to assess photocatalyst decomposition subjected to HER. Through X-ray photoelectron spectroscopy (XPS), a formula of (MoS_2_)_82.99_Ag_10.70_Ni_6.32_ was determined after HER, closely matching the as-prepared sample of (MoS_2_)_82.29_Ag_10.81_Ni_6.91_ (details in Table S1†). Both agree well with the nominal stoichiometric compositions within fitting errors. This suggests that neither Ni nor Ag are dissociated from the MoS_2_ matrix after photo irradiation.

**Fig. 4:**
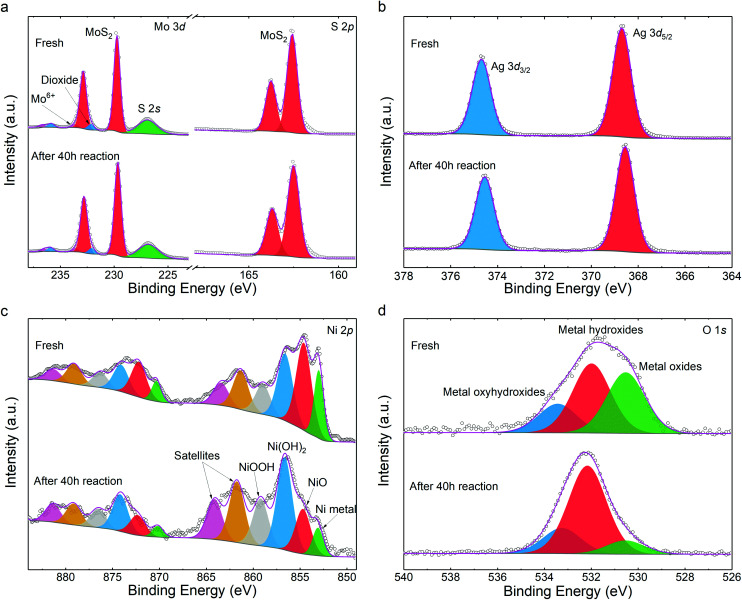
XPS spectra of the photocatalyst before and after HER: (a) Mo 3*d* and S 2*p*, (b) Ag 3*d*, (c) Ni 2*p*, (d) O 1*s*. The scatter plots are the experimental results and the grey and magenta lines are the Shirley backgrounds and fitting envelopes, respectively. Each pair of doublet peaks in panel (c) is illustrated with identical colors.

The chemical state variations of the catalysts were further investigated. The S 2*p* and Mo 3*d* peaks are unchanged (Fig. 4a) and comparable to those of pristine MoS_2_ (Fig. S14†). This indicates that the MoS_2_ host is chemically stable during the synthetic and photocatalytic processes, in line with other noble metal-decorated MoS_2_ composites.^30^ As the buffer between MoS_2_ and nickel NPs, the silver 3*d* states keep the same spectroscopic signature. As shown in Fig. 4b, the peaks at 368.63 eV and 374.70 eV are assigned to Ag 3*d*_7/2_ and 3*d*_5/2_. The energies denote that the Ag chemical state is between those of the Ag_2_S and Ag^0^ species.^48^ In conjunction with the HRTEM determination from Fig. 1h, it indicates that the partially reduced silver acts as a stable path for charge carriers during photocatalysis. Such stability is beneficial to the durability of the catalyst. Fig. 4c illustrates that the Ni metal and NiO were partially oxidized during the HER. Compared to the fresh sample, more Ni(OH)_2_ and NiOOH are found as products of oxidization.^49,50^ The increase in –OH content is also visible in the O 1*s* spectra in Fig. 4d.

**Fig. 5:**
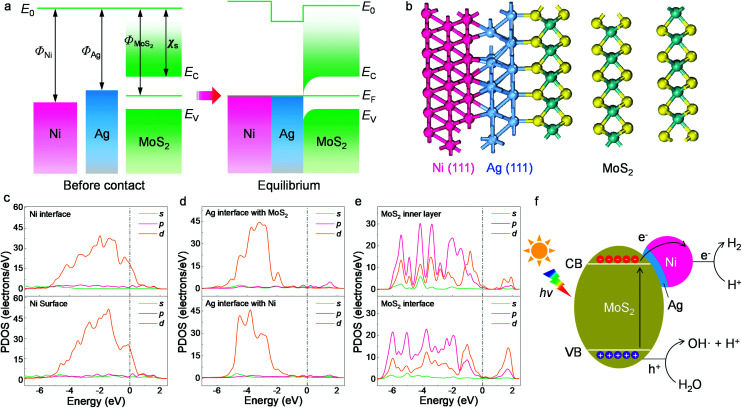
Structural and electronic computation results. (a) Band alignment of the ternary interface. *E*_0_, *E*_F_, *E*_C_, *E*_V_, *Φ*_Ni_, *Φ*_Ag_ and *χ*_s_ represent the vacuum level, Fermi level, the conduction and valence bands of MoS_2_, the work functions of Ni and Ag and the electron affinity of MoS_2_, respectively. (b) Optimized structure in side view. (c) PDOS of the Ni (111) surface and the interface with Ag. (d) PDOS of the Ag (111) interfaces with MoS_2_ and Ni. (e) PDOS of the MoS_2_ inner layer and interface. The upper and lower graphs in each panel have the same PDOS scale. (f) Illustration of the photocatalytic hydrogen evolution process.

Possible photocatalytic mechanisms were investigated and are discussed for the HER occurring on the present composites. Based on the microstructural and spectroscopic results, we first propose the charge transfer mechanism within the designed heterojunctions. The band alignment is depicted in Fig. 5a, where the Ni nanoparticles, Ag buffer, and MoS_2_ are arranged according to the TEM determinations from Fig. 1. The work function values of Ni (111) and Ag (111) are 5.35 eV and 4.74 eV, respectively.^51^ As for MoS_2_, its electron affinity is 4.23 eV and its work function is 5.20 eV, as obtained from 12-layer samples after annealing.^52,53^ Before contact, the metals and MoS_2_ have equivalent vacuum levels but different Fermi energy values. When they are joined together, electrons tend to move from Ag towards Ni and MoS_2_ due to the work function differences and an equilibrium state is then achieved, leading to a band-bending feature with a narrow anti-barrier layer. Once the ternary material is under illumination, photoexcited electrons migrate from MoS_2_ to the metal side and finally reach the Ni surface. The electrons participate in photocatalytic water splitting or photodegradation and are immediately consumed rather than forming a stable built-in electric field. Replacing Ag^+^ with the more oxidative Au^3+^ ion during the reaction fails to create surface contact due to both the fast redox reaction of Au^3+^ with MoS_2_ and Ni and the mismatch between the Au (111) lattice and the MoS_2_ basal plane.^31^

The proposed band alignment is further explicated by first-principles calculations. Here, Ag (111) is employed as a model buffer layer following the morphological determination of the basal attachment. The optimized structure of the Ni_75_Ag_32_(MoS_2_)_64_ supercell in Fig. 5b shows that Ag (111) can be stably intercalated between the MoS_2_ basal plane and the Ni particles. After adsorbing Ag and Ni atoms, the relaxation is negligible at the MoS_2_ layers, despite a slight deformation of the surface exposed to the Ni (111) site (Fig. S15†). The Ni–Ag–MoS_2_ hybrids are interatomically bonded, with Ag–S and Ag–Ni bond lengths of ∼2.77 Å and ∼2.63 Å, respectively. Charge transfer happens through Ag–S and Ni–Ag, as denoted by the increased values of the partial density of states (PDOS) at the Ni surface in Fig. 5c and by the electron density difference results (Fig. S16†). This leads to an overall Ag chemical state between Ag_2_S and Ag^0^ that agrees with the XPS results in Fig. 4b. The delocalized electrons tend to accumulate throughout the Ni surface, as shown by the PDOS plot.

Despite the electronegativity value of Ag (1.93) being slightly higher than that of the transition metal Ni (1.91),^51^ electron migration from the Ag buffer to Ni is favored due to the significant number of dangling bonds on the Ni surface able to host migrated electrons (Fig. S16†). In addition, the large fraction of Ni in the synthesized system further amplifies the quantity of the dangling bonds as hosts for migrated electrons. The whole system is also stabilized due to the perfect lattice matching. The MoS_2_ host switches from semiconducting to metallic at the interfacial region according to the PDOS results in Fig. 5c–e. Unlike the subtly changed MoS_2_ DOS, the *d*-DOS from Ni disperses across the bandgap of the host. It acts as a dangling bond that enables free electron migration from the semiconductor to the metal part, leading to a substantial decrease in interfacial resistivity (Fig. 2c). Careful investigation of the PDOS shows that the partially filled d-state of Ni overlaps with unoccupied states next to the valence band maximum (VBM) of MoS_2_. As a result, this reduces the final DOS volume proportional to the optical transition, leading to the smaller excitonic peak B shown in Fig. 3c.

Based on the experimental and first-principles results, the photocatalytic mechanism is proposed and depicted in Fig. 5f. The photoexcited electrons from the valence bands (VBs) of the layered MoS_2_ swiftly migrate to the metal parts due to the intrinsic dangling bonds in the *d*-orbit of the nickel, leaving positive holes (h^+^) in the VBs of the matrix. The created electrons accumulate in the Ni part. Such an efficient separation is not feasible using only the Ag buffer due to the rather low DOS overlap, which is proved in the comparison experiment. Recombination between the electrons and holes is delayed in the multilayer MoS_2_ host due to the indirect bandgap and an energy dip at the M/S interface (Fig. 5a). The energy of the h^+^ is slightly larger than the water oxidization energy and thus it is able to oxidize H_2_O to H^+^ and OH. The H^+^ ions are reduced to H· by the migrated electrons at the Ni surface (electron reservoir). Later, H_2_ molecules are formed and released from the suspension. This process is specifically favored on the Ni atoms, which have a large amount of charge accumulation (Fig. S16†) for water splitting, as reported previously.^31,54,55^ As the other products from water oxidization, OH radicals can react with the photocatalysts and combine with each other to produce peroxide water, which can further decompose to water and O_2_ molecules. The clear signatures of the NiOOH and Ni(OH)_2_ contents on the NiNP surfaces (Fig. 4) show that part of the NiNP surface is (hydro)oxidized, as a typical product following the annexation of OH onto the nickel metal. The oxygen evolution reaction (OER) catalysts of nickel hydroxides^32,56^ further interact with the subsequent OH radicals. In addition, the multi-step OER process is shortened because the hydroxyls can directly interact with the OER catalyst without needing the overpotential of water oxidization (*e.g.*, by electrochemical processes) to create them.^56,57^ As a result, an increase in oxygen content is observed along with the appearance of hydrogen gas in the catalytic reactor after visible light irradiation for a long time (Fig. S17†). In summary, the high H_2_ production is attributed to efficient electron migration *via* Ni *d*-state tunneling across the silver structural stabilizer after the photoexcitation of the electrons from the activated MoS_2_ basal planes with the indirect bandgap.

## Experimental

### Materials and synthesis

MoS_2_ flake powders (>99.5%, Nanjing Emperor Nano Material Co. Ltd) and nickel nanopowder (Ningxia Orient Tantalum Industry, Co. Ltd) with an average diameter of 200 nm were weighed and mixed in a DURAN Erlenmeyer flask (125 mL). Ultrapure water (75 mL, 18.2 MΩ cm at 25 °C) was then added to the flask. Vigorous vibration was performed to evenly distribute the powder. Silver nitrite (0.01 mol L^−1^, Sigma-Aldrich) was then added, and the flask was sealed with ambient air inside. The mixed suspension underwent wet chemical synthesis in an ultrasonic cleaner (35 kHz, 70 °C) for 4 hours. A typical weight percentage is MoS_2 _: Ni : Ag = 84 : 6 : 10. After synthesis, samples were kept in the ultrasonic cleaner and cooled to room temperature. The remaining water was removed and the catalyst samples were washed with ultrapure water before the characterizations or HER tests.

### Characterizations

The catalyst in aqueous solution was deposited onto either silicon substrates for SEM (ZEISS Sigma FESEM) or lacey carbon film (Agar Scientific Ltd) for TEM (JEOL JEM-2200FS EFTEM). SEM-EDS and TEM-EDS analyses were also carried out to verify the elemental distribution. During the TEM tests, a JEOL EM-21010/21311HTR high tilt holder was used to allow high tilt angles up to ±80°. This is helpful to verify whether the NiNPs located on the basal planes of MoS_2_.

*I*–*V* measurements were recorded through a combined SEM-AFM technique. The substrates were prepared by depositing 30 nm Au coating on a silicon surface. Samples were deposited on the Au surface. After drying, the Au surface was bridged to a conductive steel plate using silver paint. Tape exfoliation was not applicable here due to the weak adhesion between the Au coating and the Si surface. A sample was first verified under SEM, with its position marked. After that, the sample was transferred to the AFM (Veeco Dimension 3100). The morphologies were obtained under the tapping mode with a NSC14/Al tip (MikroMasch). *I*–*V* measurements were then taken under the force mode with a CSC17/Pt tip (MikroMasch) with conductive Pt coating on both sides. The voltage applied ranged from −8 V to 8 V.

The optical absorbance property was measured by a Shimadzu UV-2600 spectrophotometer. Both the photocatalyst and pure MoS_2_ samples were in aqueous suspension in quartz cuvettes at the same concentration (0.4 g L^−1^). A Thermo Fisher Scientific ESCALAB 250Xi XPS system with an Al K_α_ X-ray source was used to analyze the chemical state variations. The XPS spectra were calibrated with the reference value of adventitious C 1*s* at 284.8 eV and then deconvoluted with the Thermo Scientific Avantage Software. The specific surface area of the photocatalyst was measured by a Micromeritics ASAP 2020 analyzer. The catalyst in suspension was sand-bathed overnight to remove most of the water and then heated to 300 °C (10 °C min^−1^) for 4 hours in a N_2_ atmosphere (100 mL min^−1^).

### HER measurements

The catalyst (5 mg) was dissolved in ultrapure water (30 mL) in a quartz bottle. HER tests were carried out with a PCX50B multi-channel system (Beijing PerfectLight Technology, Co., Ltd). The reaction proceeded under top illumination from LED lamps (0.495 W, white light). The spectral irradiance of the lamps was measured by a THORLABS PM100D optical power meter with a S310C thermal power sensor. A cutoff design ensured the elimination of wavelengths under 420 nm. The HER proceeded at room temperature. During the tests, magnetic stirring (120 rpm) was employed to ensure uniform light illumination. The gas was analyzed on an Agilent 490 Micro GC that was carefully calibrated for hydrogen quantity. Ar was used as a carrier gas. The H_2_ evolution reaction as well as the gas chromatography measurements were performed without specific removal of the residual air in the photocatalytic system. Thus, precise monitoring of the variation of O_2_ concentration was not applicable. A preliminary HER test carried out under the air/Ar mixture (Fig. S17†) shows simultaneous increase in O_2_ and H_2_, indicating overall water splitting. The quantum efficiency was determined by the number of absorbed photons and photoexcited electrons per unit time. LED lamps with an approximately monochromatic wavelength were used as light sources.

### Computational details

The composite structure consists of layered MoS_2_ as well as Ag and Ni nanoparticles. It was observed that the catalytic ability of the ternary compounds was only active under radiation of incident photon energy larger than the layered MoS_2_ bandgap (Fig. 3c). This is due to the band structure properties provided by edge contact after metallization.^31^ Here, we emphasize on computations using the basal contact where Ag (111) sits between the MoS_2_ surface and the NiNPs (Fig. 1d–g). To fit the experimentally determined microstructure, the simplified supercell contains four layers of MoS_2_, two layers of Ag and three layers of Ni. Ag (111) serves as an interatomic interlayer that bonds with both MoS_2_ and the Ni nanoparticles. The optimized lattice parameters of the corresponding heterojunction structure with a unit cell of reconstructed layers were *a* = *b* = 12.63 Å and *α* = *β* = 90°, *γ* = 120°. The full model of this slab has a supercell of Ni_75_Ag_32_(MoS_2_)_64_. During simulations, the top two MoS_2_ layers were allowed to be optimized and the two lowest layers were fixed at bulk positions. A vacuum region of 15 Å was selected in the *z*-direction to exclude mirror interactions between neighboring images.

All calculations were performed under the framework of unrestricted spin-polarized density functional theory implemented in the CASTEP package.^58^ The exchange–correlation functional was treated as a Perdew–Burke–Ernzerhof (PBE) functional within a generalized gradient approximation (GGA),^59^ which leads to an underestimation of the bandgap values compared with the experimental results.^60,61^ The plane-wave cutoff energy was set to 300 eV. LDA+U formalism was used for the Ni, Ag, and Mo elements for electronic structure calculations. The effect of van der Waals interactions was introduced explicitly through the empirical correction scheme proposed by Grimme.^62^ The Brillouin zone integrations were carried out on a 2 × 2 × 1 Monkhorst–Pack *k*-point grid in the geometry optimizations and a 5 × 5 × 1 *k*-point grid in the band structure and DOS calculations. Considering the lowest spin state of the system, the initial value for the number of unpaired electrons of the Ni–Ag–MoS_2_ system was set as 100 in the spin-polarized calculation.

## Conclusions

In summary, we successfully realized a sustainable, efficient, and universal water splitting strategy using only visible light, water, and novel ternary photocatalysts. A high rate of 73 μmol g^−1^ W^−1^ h^−1^ of hydrogen production was achieved, which is promising for practical hydrogen production. The Ni–Ag–MoS_2_ composite uniquely facilitates photocatalytic hydrogen evolution due to the lattice matching between Ag and the MoS_2_ basal plane and the dangling bonds in the *d*-orbitals of Ni. The dream of ultimate clean fuel production is closer. Besides providing the synthetic routes and physical mechanisms, this work serves as a debut of a modern photocatalyst aiming for simultaneous decontamination and fuel production.

## Conflicts of interest

There are no conflicts to declare.

## Supplementary Material

NR-014-D2NR01489K-s001
