# Supplementary material for: Nickel nanoparticle-activated MoS2 for efficient visible light photocatalytic hydrogen evolution
Source: Nanoscale. 2022 Apr 28;14(24):8601–10. doi: 10.1039/d2nr01489k (PMC9219418; doi:10.1039/d2nr01489k)
Supplement: NR-014-D2NR01489K-s001 [file NR-014-D2NR01489K-s001.pdf]

Electronic Supplementary Material (ESI) for Nanoscale.
This journal is © The Royal Society of Chemistry 2022

Supporting Information

Ni nanoparticle activated MoS_2 for efficient visible-light photocatalytic hydrogen evolution

Xinying Shi[a,b], Meng Zhang[c,*], Xiao Wang[c], Andrey A. Kistanov[a,*], Taohai Li[a,d], Wei Cao[a], and Marko Huttula[a]

[a] Nano and Molecular Systems Research Unit, University of Oulu, P.O. Box 3000, FI-90014, Oulu, Finland.

[b] School of Physics and Electronic Engineering, Jiangsu Normal University, Xuzhou 221116, China

[c] Department of Physics, East China University of Science and Technology, Shanghai 200237, China

[d] College of Chemistry, Key Lab of Environment Friendly Chemistry and Application in Ministry of Education, Xiangtan University, Xiangtan 411105, China

[*]Corresponding authors: mzhang@ecust.edu.cn (M.Z.); andrey.kistanov@oulu.fi (A. A. K.)

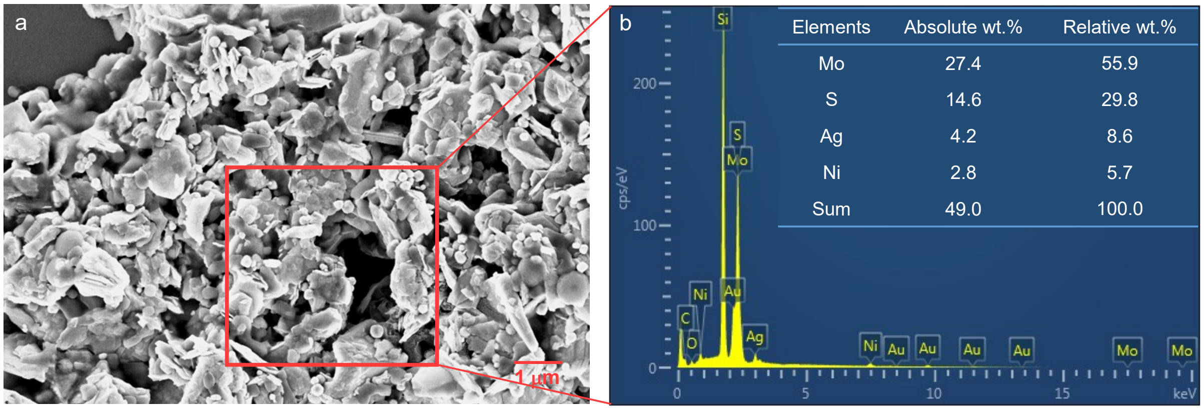

Figure S1. SEM images of synthesized composites. (a) SEM image of the sample with nominal composition of $(MoS_2)_{84}Ag_{10}Ni_6$. (b) Overall EDS area analysis and elemental quantification of Mo, S, Ag and Ni.

Quantifying Mo and S elements is difficult since the emission energies of Mo L_α (2.292 keV) and S K_α (2.309 keV) are very close to each other. But the sum of Mo and S is reliable to quantify the amount of MoS_2. To prepare the substrate for both SEM and conductive-AFM, silicon plate was coated with Au (20 nm thick). Synthesized composites in aqueous suspension were then dripped on the surface of Au coating. Therefore, strong signals of Si and Au appeared in the spectrum. Without consideration of Si, Au, O and C elements, the relative weight percentages of MoS_2, Ag and Ni are 85.7%, 8.6% and 5.7%, respectively. It agrees well with the stoichiometric ratio of $(MoS_2)_{84}Ag_{10}Ni_6$.

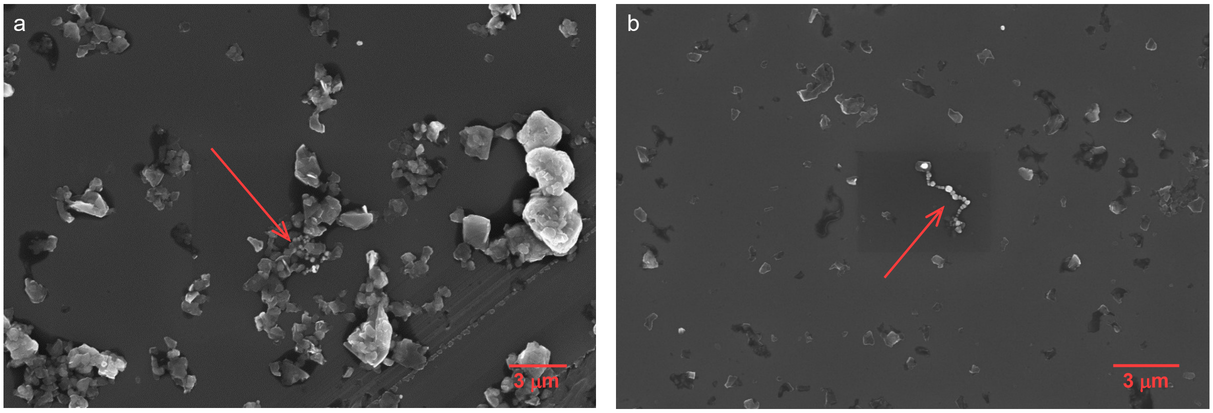

Figure S2. SEM images of control group samples. (a) Very few NiNPs appear around MoS_2. (b) Most NiNPs aggregate together rather than joining to the MoS_2 flakes.

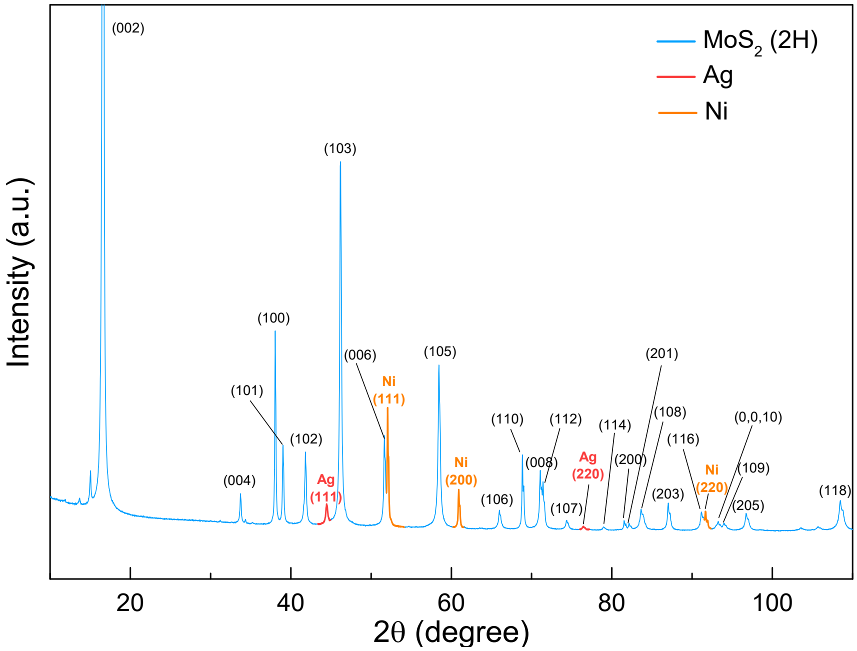

Figure S3. XRD pattern of the photocatalyst. The blue, red and orange lines show the patterns of MoS_2, Ag and Ni, respectively.

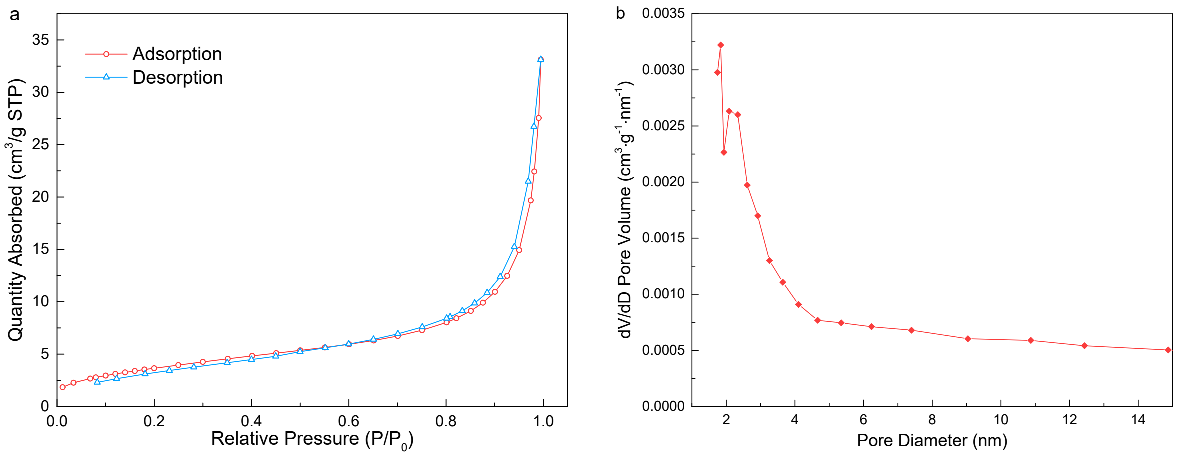

Figure S4. Specific surface area measurements. (a) Nitrogen adsorption-desorption isotherm linear plot; (b) Barrett-Joyner-Halenda (BJH) adsorption pore distribution. Panel (a) shows the nitrogen adsorption–desorption curve of the synthesized photocatalyst, indicating the presence of nanopores. The photocatalyst has a Brunauer–Emmett–Teller (BET) surface area of 13.6231 $m^2 \cdot g$. The pore size mainly distributed around 2 ~ 3 nm.

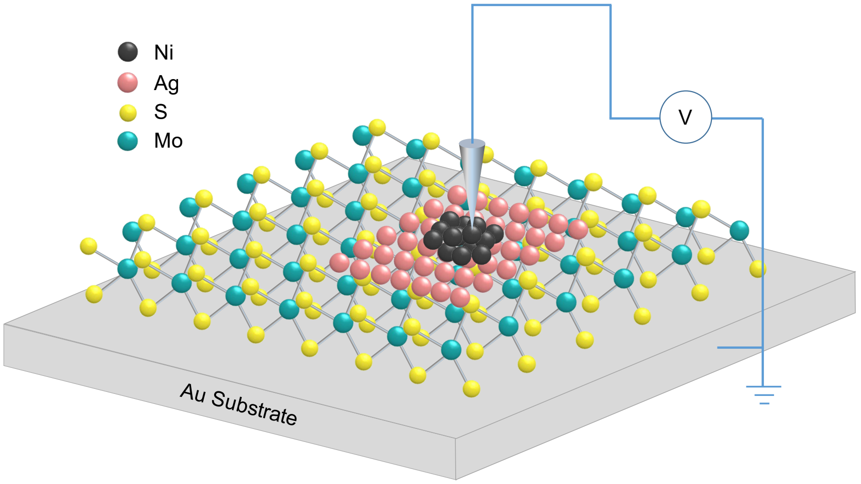

Figure S5. Schematic diagram of *I-V* measurement via C-AFM method. Synthesized composites are deposited on Au film. Both the front and back side of the AFM tip have conductive Pt coating.

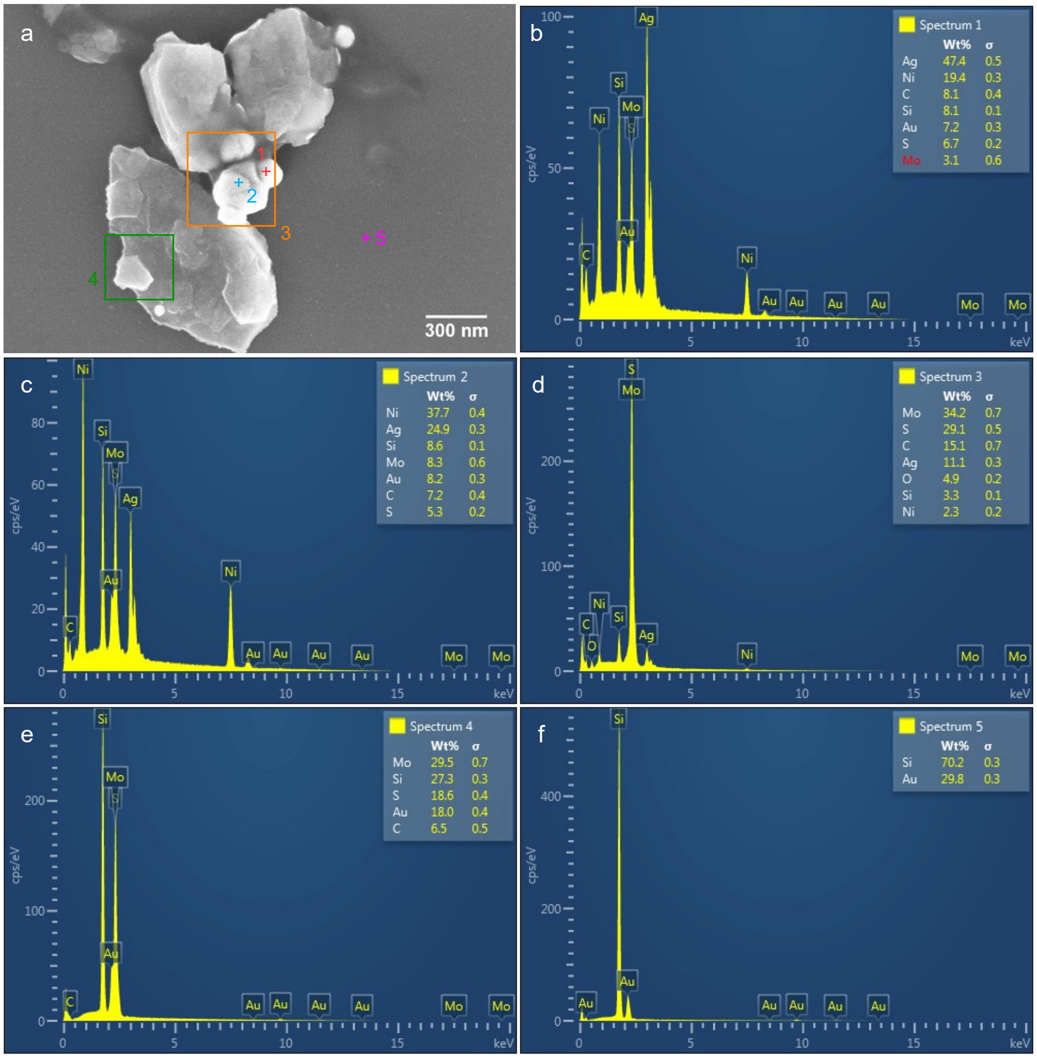

Figure S6. Elemental analysis through EDS. (a) SEM image. (b) Point analysis 1 (Ag). (c) Point analysis 2 (Ni). (d) Area analysis 3 (interfacial region). (e) Area analysis 4 (MoS_2 flake). (f) Point analysis 5 (Au coating on silicon substrate). The cross marks in panel (a) show the positions of point analysis. Si and Au signals appeared in all the elemental analysis spectra originating from the substrates.

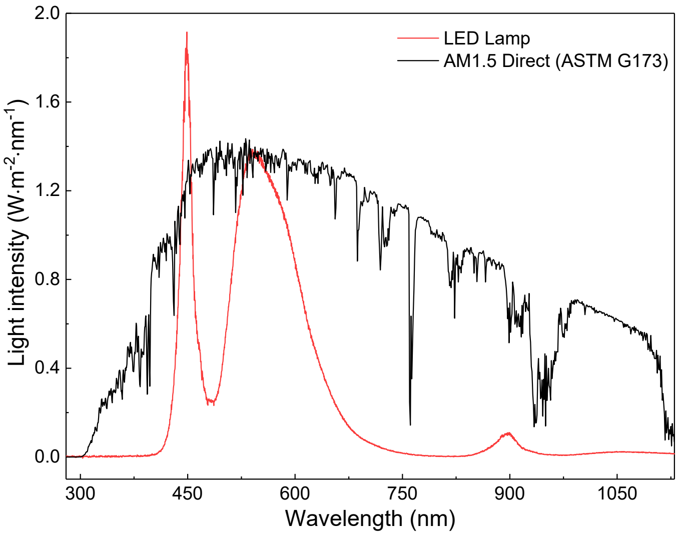

Figure S7. Spectrum of LED lamp in comparison with solar radiation (AM1.5).

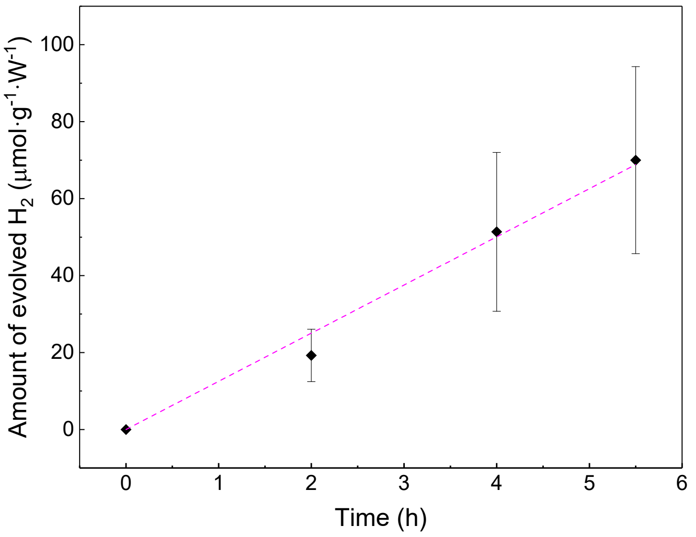

Figure S8. HER tests with the catalyst of MoS_2-Au-Ni.

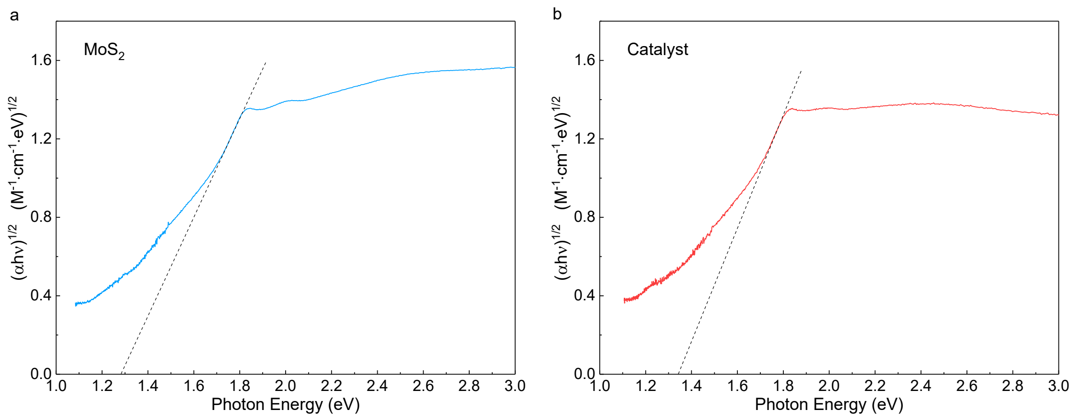

Figure S9. Determination of absorption band gaps through absorbance spectra. (a) MoS_2. (b) Catalyst.

The absorption bandgap can be extrapolated with the relation: $(\alpha h\nu)^{1/m} \propto (h\nu - E_g)$, where α, $h\nu$, E_g and m are the absorption coefficient, photon energy, bandgap, and an exponent related to the optical transition types ($m = 2$ for indirect allowed transition while $m = 1/2$ for direct allowed transition). In this work, optical absorbance measurements were performed with samples in aqueous suspension in quartz cuvettes. Therefore, the absorption coefficient α can be calculated by the relation: $\alpha = A/(b \cdot c)$, where A, b and c are the absorbance (arbitrary unit), length of cuvettes (cm) and molar concentration (mol/L or M), respectively. In this calculation, the absorbance was normalized, by setting the value at transition point as 100%.

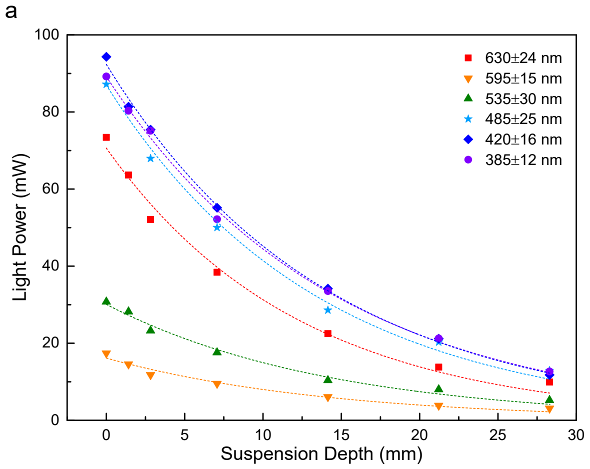

b

Wavelength (nm)	A	B
385 ± 12	89.18501	-0.06962
420 ± 16	92.35364	-0.07143
485 ± 25	86.98943	-0.07416
535 ± 30	30.08033	-0.06988
595 ± 15	16.16145	-0.07031
630 ± 24	70.63453	-0.08138

$P_i = A \cdot e^{Bx}$

Figure S10. Light power attenuation by the photocatalyst in aqua suspension with magnetic stirring. (a) Attenuation of incident light of different wavelength. The patterns are experimental values and the dot lines are fitted with exponential functions. (b) List of fitted attenuation correlation functions which have very close exponential coefficients (B) and show good conformity with Beer-Lambert Law. P_{i} represents the measured light power of different wavelength at certain suspension depth.

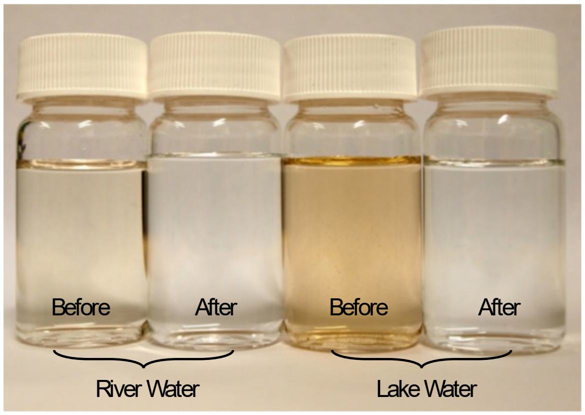

Figure S11. Decoloration of river water and lake water.

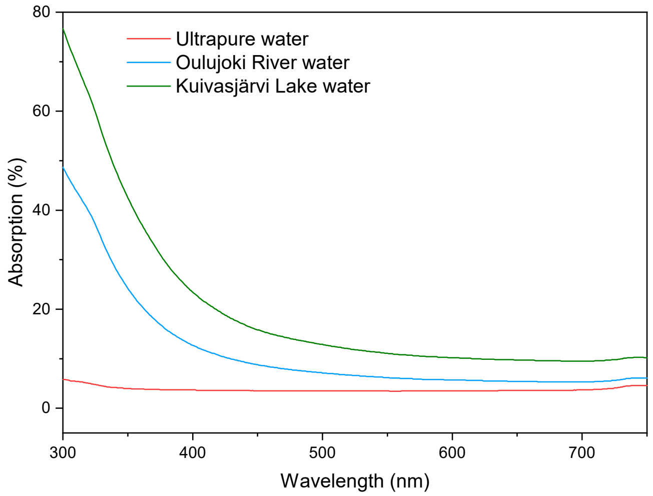

Figure S12. Visible light absorption spectra of ultrapure and natural water samples.

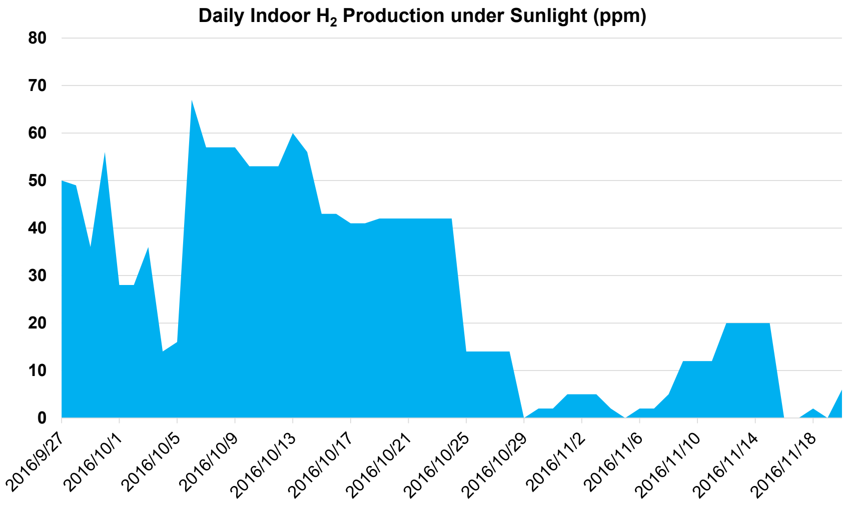

Figure S13. Daily indoor H_2 production under natural sunlight.

The catalyst (5 mg) is dispersed in 50 mL distilled water in a SCHOTT DURAN Erlenmeyer flask. During the daily tests, the flask was put on a table next to a double glazed window in Oulu, Finland, and the H_2 concentration was measured with a portable H_2 alert detector.

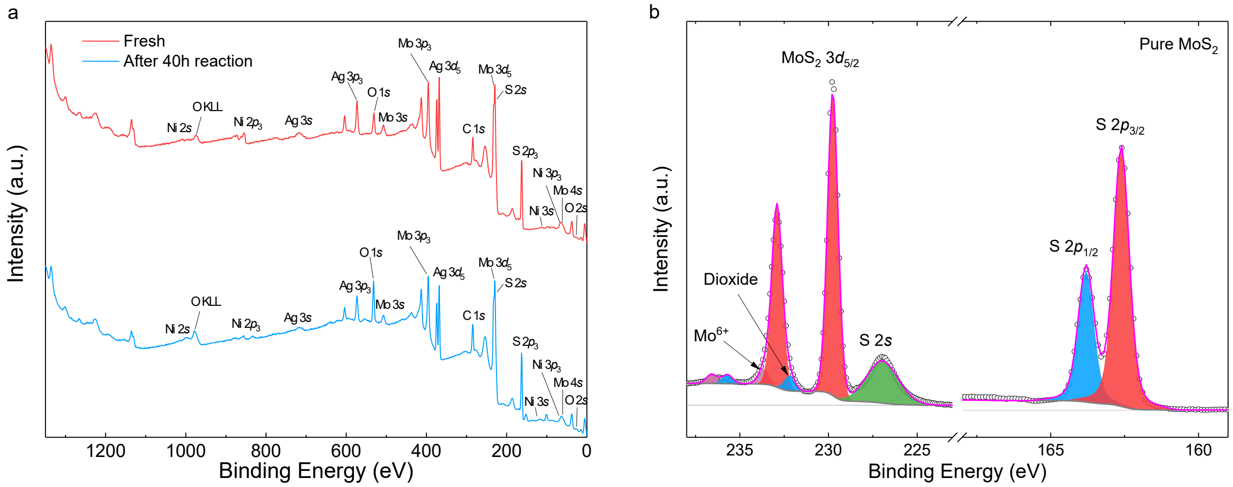

Figure S14. (a) XPS survey scans of fresh and used photocatalysts. (b) Mo 3*d* and S 2*s* spectra of commercial MoS_2 flakes.

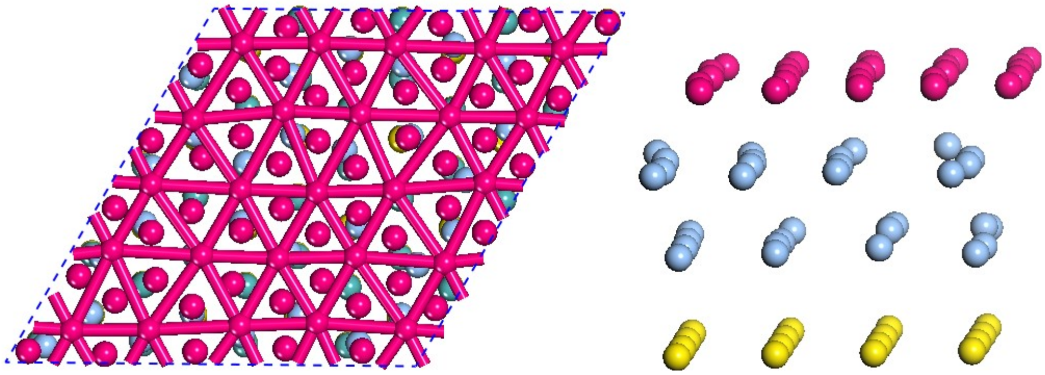

Figure S15. (a) Top view of DFT-optimized crystal structure of exposed reactive surface Ni (111) of Ni-Ag-MoS_2 nanosheets. (b) Side view of structure of hetero-interface. The red, blue and yellow balls represent the Ni, Ag, and S atoms, respectively.

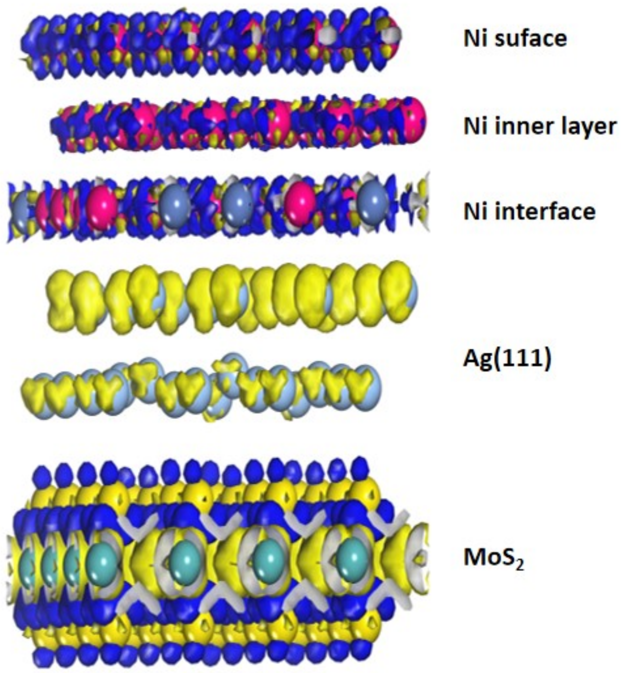

Figure S16. Side view of calculated deformation electron density of the Ni-Ag-MoS_2 nanosheets. Yellow region represents charge depletion, whereas the blue region indicates the charge accumulation in the system. The surface isovalue for electron density is 0.03 $e^-/Å^3$.

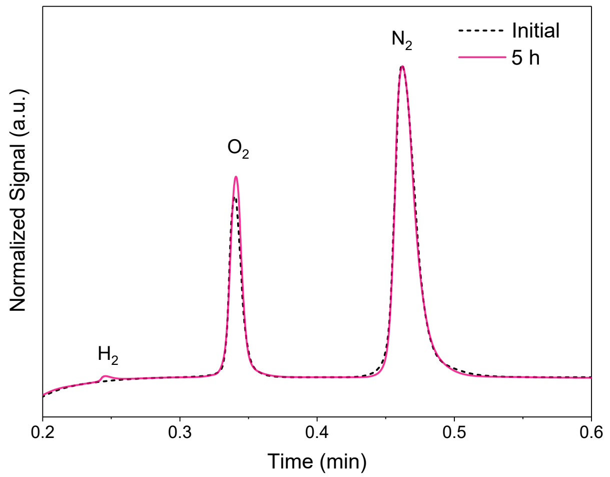

Figure S17. Gas chromatography results. The scatter plot is measured before HER and the plot in red is measured after 5h HER. The two spectra were normalized to make identical intensity of the N_2 peaks. Apparent increase of the oxygen intensity denotes the existence of the OER with the HER.

Table S1. Peak fitting details of Ni 2*p*, O 1*s*, Ag 3*d*, Mo 3*d*, and S 2*s* XPS spectra. All percentage values are calculated after eliminating of adventitious C 1*s*, substrate-induced Si 2*p*, and S 2*s*.

Valence states	Samples	Components	Binding Energy (eV)	FWHM (eV)	Atomic Percentage (%)			wt.%
					Oxygen included	Oxygen excluded		Oxygen excluded
Ni $2p_{3/2}$	Fresh	Ni metal	852.91	1.25	0.82	0.97	6.62	6.91
		NiO	854.51	2.00	1.71	2.02		
		$Ni(OH)_2$	856.51	2.00	1.30	1.53		
		NiOOH	858.89	2.00	0.58	0.68		
		Satellite 1	861.25	2.00	0.77	0.91		
		Satellite 2	863.35	2.00	0.43	0.51		
	Used	Ni metal	853.00	1.35	0.29	0.38	6.01	6.32
		NiO	854.60	2.00	0.67	0.90		
		$Ni(OH)_2$	856.55	2.00	1.34	1.79		
		NiOOH	859.12	2.00	0.72	0.96		
		Satellite 1	861.66	2.00	0.91	1.21		
		Satellite 2	864.05	2.00	0.57	0.77		
O 1*s*	Fresh	Metal oxides	530.50	2.00	5.90	/		/
		Metal hydroxides	531.96	2.00	6.73			
		Metal oxyhydroxides	533.41	2.00	2.80			
	Used	Metal oxides	530.52	2.00	2.72			
		Metal hydroxides	532.14	2.00	17.51			
		Metal oxyhydroxides	533.23	2.00	5.09			
Ag $3d_{5/2}$	Fresh	$Ag^{(0\sim1)}$	368.63	0.88	4.76	5.63		10.81
	Used	$Ag^{(0\sim1)}$	368.57	0.89	4.13	5.53		10.70
Mo $3d_{5/2}$	Fresh	Mo^{4+} (MoS_2)	229.70	0.73	21.91	25.91	28.48	48.59
		Mo^{4+} (dioxide)	232.14	0.73	1.34	1.58		
		Mo^{6+}	233.61	0.73	0.84	0.99		
	Used	Mo^{4+} (MoS_2)	229.62	0.73	18.94	25.36	28.17	48.43
		Mo^{4+} (dioxide)	232.07	0.73	1.24	1.66		
		Mo^{6+}	233.51	0.73	0.86	1.15		
S $2p_{3/2}$	Fresh	MoS_2	162.53	0.80	50.13	59.27		33.70
	Used	MoS_2	162.46	0.80	45.03	60.29		34.55
